# Is the Modified Distress Thermometer Useful for Screening Pregnant Women With COVID-19 for Psychological Distress?

**DOI:** 10.7759/cureus.22878

**Published:** 2022-03-05

**Authors:** Sherif Mohamed, Rabia Shaukat

**Affiliations:** 1 Department of Pulmonary Medicine, Faculty of Medicine, Assiut University, Assiut, EGY; 2 Department of Medicine, Sultan Bin Abdulaziz Humanitarian City, Riyadh, SAU; 3 Department of Family and Community Medicine, University of Texas (UT) Health Science Center, Houston, USA

**Keywords:** psychological, pregnancy, screening, modified distress thermometer, covid-19

## Abstract

Background: Pregnant women may be more vulnerable than others to the psychological and social effects of the coronavirus disease 2019 (COVID-19) pandemic. In this study, we try to answer the question - is the modified distress thermometer (m-DT) useful for screening pregnant women with COVID-19 for psychological distress?

Methods: We have used the m-DT to screen pregnant women with COVID-19 for psychological distress. A total of 112 pregnant women with COVID-19 were prospectively enrolled. The study participants were asked to rate their distress in the past three days on an 11-point visual analog scale ranging from 0 (no distress) to 10 (extreme distress). They were then asked to fill in the problem list (PL) which accompanied the visual image of the m-DT. To explore the association between these scores and the clinical variables, binary logistic regression tests were carried out.

Results: Sixty-eight percent (76/112) of the study subjects experienced significant (m-DT score ≥ 4) COVID-19-related distress. Regression analysis showed that m-DT score of ≥4 had statistically significant associations with gravida status length of quarantine time, the presence of chronic medical or respiratory disease, fears, worry, shortness of breath, and sleep. Multivariable analysis confirmed that the presence of chronic respiratory disease, shortness of breath, and sleep were independent factors associated with significant distress in pregnant women with COVID-19.

Conclusion: With the use of m-DT, two-thirds of pregnant women with COVID-19 experienced significant distress. This distress was significantly related to older age, multigravida, exposure to longer quarantine time, presence of underlying medical disorder, and the presence of chronic respiratory disorders. The presence of chronic respiratory disease, shortness of breath, and sleep disturbance were independent factors associated with significant distress in pregnant women with COVID-19.

## Introduction

The coronavirus disease 2019 (COVID-19) affected virtually all countries and had posed a significant threat to the health of the global population, with considerable direct and indirect psychological and social effects [1,2]. Some groups, like pregnant women, may be more vulnerable than others to the psychosocial effects of the pandemic [3,4]. Additional risk factors for mental health affection include the drawbacks of the economic downturn, as well as the consequences of quarantine and its closely associated social and physical distancing [2,5,6]. Notably, COVID-19 has resulted in an increase in symptoms of various psychiatric disorders like depression, anxiety, post-traumatic stress disorder (PTSD), and obsessive-compulsive disorder (OCD) [7,8]. Because of COVID-19, healthcare deliveries across the world had to be modified to accommodate COVID-19 guidelines, which put added stress on the health care system [9,10]. Moreover, a lot of panics and increased anxiety across the globe have occurred recently after an announcement by the WHO about the COVID-19 variant of concern or Omicron [11]. 

The importance of screening cancer patients for psychological distress using valuable tools, like distress thermometer (DT) is well established [12,13]. This had led us to the idea of adopting a modified version of this DT (m-DT) to screen adults with suspected or confirmed COVID-19 for psychological distress [14]. With the m-DT, we have seen that 60% of adult Egyptian COVID-19 patients experienced significant distress [14]. Therefore, in the current study, we aimed to evaluate the utility of this modified DT (m-DT) as a screening tool for evaluating psychological distress among pregnant women with COVID-19.

## Materials and methods

Study population

Enrollment included Egyptian pregnant women who fulfilled the national criteria of suspected or confirmed cases of COVID-19 and managed as outpatients or admitted at the obstetrics and gynecology department of a university hospital in isolation rooms with an adequate command of speaking and reading the Arabic language were prospectively enrolled in the current study [15]. Subjects with a history of or undergoing current treatment for psychiatric illness and those who did not consent to participate in the study were excluded. Standard sociodemographic data of the enrolled subjects were collected including age, education level, and occupation. Medical records were reviewed for obstetric history, past medical history, vaccination history, history of recent travel, and history of quarantine. 

The study objectives and procedures were fully explained to eligible subjects. The necessary infection prevention and control measures for handling patients with COVID-19 were undertaken and the person carrying the questionnaire wore full personal protective equipment (PPE) [15]. Ethical approval was obtained from the local institutional review board. Written consent has been obtained from the subjects for carrying out the study procedures.

Modified distress thermometer (m-DT)

**Figure 1:**
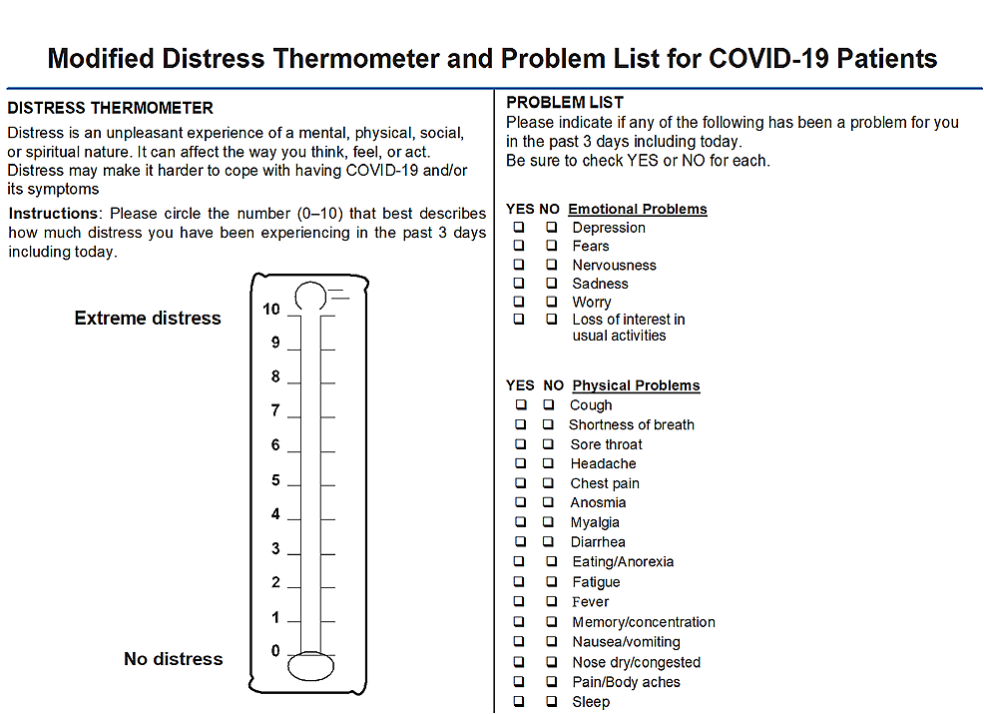
Modified distress thermometer and problem list COVID-19: coronavirus disease 2019 The image is obtained with permission from Mohamed et al. [14].

As shown in our recent publication [14], we have utilized the m-DT to screen the enrolled pregnant women for psychological distress, with the use of a cutoff score of ≥4 for significant distress [12,14]. The patients were screened at their first outpatient/emergency department visit or inpatient admission. As the m-DT includes an 11-point visual analog scale ranging from 0 (no distress) to 10 (extreme distress), the study subjects were asked to rate their distress in the past three days on this scale (Figure 1) [14].

Patients were then asked to fill in the problem list (PL) which accompanied the visual image of the m-DT. This implies that they have to check, whether or not (yes/no), during the previous three days, they have any of the problems listed (Figure 1). A research assistant helped those illiterate patients to rate their distress and fill in the PL. Correlation between the PL and m-DT was carried out to identify the nature of distress and related factors.

Statistical analysis

The following parameters were explored using descriptive statistical analysis: the frequency distribution of the m-DT, the median score, the mean score, and the standard deviation. All p-values were two-tailed. A p<0.05 was considered statistically significant. Binary logistic regression test was carried out to explore the association between the m-DT cutoff scores of 4 and the demographic and clinical variables, while binary and multivariable logistic regression tests were used to analyze the association between these scores and individual items in the PL [12,14]. We have used the Statistical Package for Social Science (SPSS) version 24 (Chicago, IL: SPSS Inc.) for data analysis.

## Results

Clinical and Sociodemographic characteristics

A total of 112 patients were prospectively enrolled. The median age was 29 (range: 19-44) years. Sixty-eight percent (76/112) of the study subjects experienced significant (m-DT score ≥ 4) COVID-19-related distress. Fifty-six percent (63/112) of patients had chronic underlying medical diseases, among which 86% (54/63) had chronic respiratory diseases. The latter included 85% (46/54) patients with asthma, 7% (4/54) with interstitial lung disease (ILD), 4% (2/54) with bronchiectasis, and 4% (2/54) with post-pulmonary tuberculosis (TB) pleuropulmonary sequelae, respectively. 

There were significant differences between those with and without significant distress with regards to age groups, gravida status, length of quarantine, presence of underlying medical disorder, and presence of chronic respiratory disorders. Table 1 shows these data.

**Table 1:** Sociodemographic characteristics of the study subjects (n=112) and their association with m-DT score ≥4* *P-value <0.05. m-DT: modified distress thermometer

Characteristic	Overall (N= 112 (%)	m-DT cut off ≥ 4 N= 76 (68%)	m-DT cutoff < 4 N= 36 (32%)	p-Value
Age in years				
Median (range)	29.0 (19.0-44.0)	-	-	0.128
Mean ± SD	29.8 ± 6.1	32.2 ± 7.2	30.7 ± 6.8	
Age groups (years)				
< 35 years	72 (64)	54 (71)	18 (50)	0.032
≥ 35 years	40 (36)	22 (29)	18 (50)	
Gravida				
Primigravida	55 (49)	32 (42)	23 (64)	0.043
Multigravida	57 (51)	44 (58)	13 (36)	
Gestational age				
< 24 weeks	63 (56)	43 (57)	20 (56)	0.919
≥ 24 weeks	49 (44)	33 (43)	16 (44)	
Educational level				
Non-educated	47 (42)	27 (35)	20 (56)	0.065
Educated	65 (58)	49 (65)	16 (44)	
Occupation				
House-wife	44 (39)	30 (39)	14 (39)	0.953
Working	68 (61)	46 (61)	22 (61)	
Length of quarantine				
< 3 months	51 (45)	28 (37)	23 (64)	0.008
> 3 months	61 (55)	48 (63)	13 (36)	
Underlying chronic disease				
Present	63 (56)	50 (66)	13 (36)	0.004
Absent	49 (44)	26 (34)	23 (64)	
Type of chronic disease				
Respiratory	54 (48)	46 (63)	8 (22)	<0.001
Non-respiratory	58 (52)	30 (37)	28 (78)	

Data from m-DT and PL analysis

The most frequent problems experienced by the study subjects and reported on the practical domain of the PL were, in descending order, worry (55.3%), shortness of breath (54.4%), depression (52.6%), chest pain (51.7%), and loss of interest, eating/anorexia, and nausea/vomiting (50.8% for each) (Table 2).

**Table 2:** The most frequent problem list items among the studied subjects (n= 112)

Problems list	No. of patients	Percentage (%)
Worry	62	55.3
Shortness of breath	61	54.4
Depression	59	52.6
Chest pain	58	51.7
Loss of interest	57	50.8
Eating/anorexia	57	50.8
Nausea/vomiting	57	50.8

Association between m-DT and the sociodemographic data and PL items

Table 3 details the association between m-DT and both the sociodemographic data and PL items. Binary logistic regression showed that m-DT score of 4 or more had statistically significant associations with eight items; gravida status length of quarantine time, the presence of chronic medical or respiratory disease, fears, worry, shortness of breath, and sleep disturbances.

**Table 3:** Logistic regression analysis for the sociodemographic variables and PL items of COVID-19 patients *P-value <0.01. **P-value <0.001. OR: odds ratio; PL: problem list; COVID-19: coronavirus disease 2019

Problem list	Item present (%)	m-DT cut-off ≥ 4 N= 76 (68%)	m-DT cut-off < 4 N= 36 (32%)	OR (95% CI)	Adjusted OR (95% CI)
Sociodemographic factors					
Age groups (≥ 35 years)	40 (36)	22 (29)	18 (50)	0.898 (0.179-0.925)	-
Gravida (multigravida)	57 (51)	44 (58)	13 (36)	2.433 (1.073-5.515)*	-
Gestational age (≥ 24 weeks)	49 (44)	33 (43)	16 (44)	0.990 (0.432-2.132)	-
Educational level (non-educated)	47 (42)	27 (36)	20 (56)	1.869 (0.988-4.764)	-
Length of quarantine (> 3 months)	61 (54)	48 (63)	13 (36)	3.033 (1.330-6.917)*	-
Chronic disease (present)	63 (56)	50 (66)	13 (36)	3.402 (1.485-7.794)**	-
Respiratory disease (present)	54 (48)	46 (61)	8 (22)	5.367 (2.159 -13.339)**	9.692 (2.232-42.090)**
Emotional problems					
Depression	59 (53)	42 (55)	17 (47)	1.381 (0.623-2.987)	-
Fears	49 (44)	39 (51)	10 (28)	2.741 (1.163-6.456)*	-
Nervousness	47 (42)	32 (42 )	15 (42)	1.018 (0.456-2.275)	-
Sadness	50 (45)	30 (39)	20 (56)	0.522 (0.234-1.162)	-
Worry	62 (55)	49 (64)	13 (36)	1.884 (0.168-0.987)*	-
Loss of interest	57 (51)	33 (43)	24 (67)	0.639 (0.195-0.987)	-
Physical problems					
Cough	56 (50)	41 (54)	15 (42)	1.450 (0.723-3.626)	6.991 (1.937-25.227)*
Shortness of breath	61 (54)	43 (56)	18 (50)	7.800 (3.168-19.094)**	-
Sore throat	50 (45)	37 (49)	13 (36)	1.679 (0.743-3.792)	-
Headache	51 (46)	36 (48)	15 (42)	1.260 (0.566-2.807)	-
Chest pain	58 (52)	40 (53)	18 (50)	0.929 (0.503-2.245)	-
Anosmia	54 (48)	32(42)	22 (61)	0.463 (0.206-1.040)	-
Myalgia	54 (48)	34 (45 )	20 (56)	0.648 (0.292 -1.432)	-
Diarrhea	43 (38)	22 (29)	21 (58)	0.988 (0.508-2.569)	-
Eating/anorexia	57 (51)	34 (45)	23 (64)	0.458 (0.202-1.035)	-
Fatigue	52 (46)	29 (38)	23 (64)	0.349 (0.153-0.794)	-
Fever	54 (48)	38 (50)	16 (44)	1.150 (0.564-2.552)	-
Memory/concentration	46 (41)	27 (36)	19 (53)	0.493 (0.220-1.103)	-
Nausea/vomiting	57 (51)	32 (42)	25 (69)	0.488 (0.217-1.097)	-
Nose dry/congested	56 (50)	31 (41)	25 (69)	0.707 (0.130-0.705)	-
Pain/body aches	49 (44)	29 (38)	20 (56)	0.594 (0.221-1.103)	-
Sleep	52 (46)	45 (59)	7 (19)	6.014 (2.341-15.452)**	5.235 (1.473-18.602)*

After adjustment to the sociodemographic and clinical characteristics, the multivariable analysis confirmed that the presence of chronic respiratory disease, shortness of breath, and sleep were independent factors associated with significant distress in COVID-19 patients. The adjusted odds ratios (95% confidence interval) for these items were 9.692 (2.232-42.090), 6.991 (1.937-25.227), and 5.235 (1.473-18.602) for the presence of chronic respiratory disease, shortness of breath, and sleep disturbances, respectively.

## Discussion

The originally adopted distress thermometer is a single-item tool that uses a point Likert scale resembling a thermometer, where the patient rates his/her level of distress over the past week [16,17], proved useful for screening cancer patients for psychological distress [12,13]. As an essential part of the assessment, a 39-item supplemental problem list (PL) of potential sources of distress was incorporated into DT to assist the provider in identifying distress [12,13,16,17].

Despite these advantages, it came to our mind that modifying this DT and its PL into a more practical and less-time consuming list of only emotional and physical items that are directly related to impacts of the COVID-19 pandemic would result in a utilizable tool for assessment of COVID-19 patients. With this modified DT, our previous work had shown a high prevalence (60%) of distress among adult COVID-19 patients [14].

In the current study, this m-DT has been used among pregnant women with COVID-19 revealed that two-thirds of the enrolled women experienced significant COVID-19-related distress. This psychological distress can be related to pregnancy itself, or COVID-19 alone. The combination of pregnancy and COVID-19 can logically explain the high prevalence of psychological distress among pregnant females [2,3,18].

Pregnancy and childbirth typically are associated with positive emotions and with motherhood. Risk factors for pregnancy-related psychopathology include unplanned pregnancy, stress associated with having common medical complications, fear of childbirth, peripartum cardiomyopathy, and pregnancy loss [18]. Despite the fact that COVID-19 has been around for more than two years, it still represents a real stressful condition, since it affects all body systems and no one is 100% immune [19,20].

The sociodemographic data showed that older age, multigravida, exposure to longer quarantine time, presence of underlying medical disorder, and the presence of chronic respiratory disorders were associated with significant distress. An m-DT cut-off score of 4 has been used in the current study. Our experience in using that cut-off score confirms that it brings the needed combination of sensitivity and specificity [14]. This is optimally needed to avoid over-misdiagnoses due to false-positive results [12-14]. Notably, nondistressed patients can be burdened with unnecessary interventions with high false-positive screening results.

The association between m-DT and both the sociodemographic data and PL items has shown interesting results. Multivariable analysis confirmed that the presence of chronic respiratory disease, shortness of breath, and sleep disturbance, were independent factors associated with significant distress in pregnant women with COVID-19. These results are in agreement with those reported for the COVID-19 pandemic [1,5], and the data we had reported previously using the m-DT [14]. Taking into consideration that chronic respiratory diseases are risk factors for severe COVID-19 disease, it seems logical that pregnant women with chronic respiratory disease and/or shortness of breath had significant COVID-19-related distress [3,21]. Sleep disturbance during COVID-19 has a multifactorial etiology and may contribute to poor quality of life, tolerance of treatment, and the development of depression [13].

Findings of the current study support those that contribute to the mental symptoms and disorders which arise during the COVID-19 pandemic to biological [22] and psychosocial factors [5,20,23,24]. Moreover, our findings confirm the importance of “early” screening of pregnant women with COVID-19 for emotional distress, using a simple and valid tool like m-DT. This could have important clinical implications. Healthcare providers should be sensitive to the distress and anxiety experienced by pregnant women with COVID-19, so that appropriate psychiatric referral could take place [3,18-23]. Furthermore, the current study could have important clinical implications. The COVID-19 pandemic may increase the risk of suicidal ideation and behavior, where suicidality related to COVID-19 may be due to the hardships imposed by the pandemic, including economic privation, side effects of the quarantine and social isolation, reduced access to general medical and mental health care, and the stigma of having COVID-19 [25]. Despite that this is a prospective study with enrolled relatively good number of pregnant women, it could not be without limitations.

Limitations

The study was carried out at a single center. Also, there is a possible convenience sampling which may affect the generalizability of the study findings to all pregnant women with COVID-19. Further studies are needed to implement screening more pregnant women for distress using m-DT.

## Conclusions

With the modified distress thermometer (m-DT), two-thirds of pregnant women with COVID-19 had significant distress. This distress was significantly related to older age, gravida status, exposure to longer quarantine time, the presence of underlying medical disorder, and the presence of chronic respiratory disorders. The presence of chronic respiratory disease, shortness of breath, and sleep disturbance were independent factors associated with significant distress in pregnant women with COVID-19. We recommend further larger studies implementing this m-DT.

## References

[1] Holmes EA, O'Connor RC, Perry VH (2020). Multidisciplinary research priorities for the COVID-19 pandemic: a call for action for mental health science. Lancet Psychiatry.

[2] Mohamed S, Elgohary G, Abd ElHaffez A (2020). Does distress thermometer have a utility in the era of COVID-19 pandemic?. Open Access Libr J.

[3] Selim M, Mohamed S, Abdo M, Abdelhaffez A (2020). Is COVID-19 similar in pregnant and non-pregnant women?. Cureus.

[4] Aghaeepour N, Ganio EA, Mcilwain D (2017). An immune clock of human pregnancy. Sci Immunol.

[5] Galea S, Merchant RM, Lurie N (2020). The mental health consequences of COVID-19 and physical distancing: the need for prevention and early intervention. JAMA Intern Med.

[6] Jain A, Bodicherla KP, Raza Q, Sahu KK (2020). Impact on mental health by “living in isolation and quarantine” during COVID-19 pandemic. J Family Med Prim Care.

[7] Jolly TS, Pandian GS, Batchelder E, Jain A (2020). Posttraumatic stress disorder exacerbation as a result of public masking in times of COVID-19. Prim Care Companion CNS Disord.

[8] Jain A, Bodicherla KP, Bashir A, Batchelder E, Jolly TS (2021). COVID-19 and obsessive-compulsive disorder: the nightmare just got real. Prim Care Companion CNS Disord.

[9] Kaur J, Jain A (2021). Challenges with long-term care placement in pediatric psychiatry during COVID-19: a case series from inpatient unit. Cureus.

[10] Jain A, Sahu KK, Mitra P (2021). Treatment of patients with mental illness amid a global COVID-19 pandemic. oronavirus Disease - COVID-19. Advances in Experimental Medicine and Biology.

[11] Jain A, Jolly TS (2021). Omicron (B.1.1.529) COVID-19 variant: a mental health perspective on lessons learned and future challenges. Prim Care Companion CNS Disord.

[12] Abd El-Aziz N, Khallaf S, Abozaid W (2020). Is it the time to implement the routine use of distress thermometer among Egyptian patients with newly diagnosed cancer?. BMC Cancer.

[13] Alosaimi FD, Abdel-Aziz N, Alsaleh K, AlSheikh R, AlSheikh R, Abdel-Warith A (2018). Validity and feasibility of the Arabic version of distress thermometer for Saudi cancer patients. PLoS One.

[14] Mohamed SA, AbdelHafeez A, Kamel E, Rashad A (2021). Utility of a modified distress thermometer in screening COVID-19 patients for psychological distress: a prospective Egyptian study. Multidiscip Respir Med.

[15] (2020). Management protocol for COVID-19 patients. https://www.researchgate.net/publication/345813633_Management_Protocol_for_COVID-19_Patients_MoHP_Protocol_for_COVID19_November_2020.

[16] Pirl WF, Fann JR, Greer JA (2014). Recommendations for the implementation of distress screening programs in cancer centers: report from the American Psychosocial Oncology Society (APOS), Association of Oncology Social Work (AOSW), and Oncology Nursing Society (ONS) joint task force. Cancer.

[17] Riba MB, Donovan KA, Andersen B (2019). Distress management, Version 3.2019, NCCN Clinical Practice Guidelines in Oncology. J Natl Compr Canc Netw.

[18] Geller PA (2004). Pregnancy as a stressful life event. CNS Spectr.

[19] Mohamed S, Saad K, Elgohary G, AbdElHaffez A, Abd El-Aziz N (2021). Is COVID-19 a systemic disease?. Coronaviruses.

[20] Yong SJ, Liu S (2021). Proposed subtypes of post-COVID-19 syndrome (or long-COVID) and their respective potential therapies. Rev Med Virol.

[21] Selim MF, Mohamed SAA, Abdou MMA, Haggag MS, Gamal Y, Abd Elhaffez AS (2020). A case of postpartum mortality due to COVID-19 infection. J Med Case Rep Case Series.

[22] Varatharaj A, Thomas N, Ellul MA (2020). Neurological and neuropsychiatric complications of COVID-19 in 153 patients: a UK-wide surveillance study. Lancet Psychiatry.

[23] Xiang YT, Jin Y, Cheung T (2020). Joint international collaboration to combat mental health challenges during the coronavirus disease 2019 pandemic. JAMA Psychiatry.

[24] Brooks SK, Webster RK, Smith LE, Woodland L, Wessely S, Greenberg N, Rubin GJ (2020). The psychological impact of quarantine and how to reduce it: rapid review of the evidence. Lancet.

[25] Laboe CW, Jain A, Bodicherla KP, Pathak M (2021). Physician suicide in the era of the COVID-19 pandemic. Cureus.

